# An Autonomous Positioning Method for Drones in GNSS Denial Scenarios Driven by Real-Scene 3D Models

**DOI:** 10.3390/s25010209

**Published:** 2025-01-02

**Authors:** Yongqiang Cui, Xue Gao, Rui Yu, Xi Chen, Dingwen Wang, Di Bai

**Affiliations:** 1 College of Electronics and Information Engineering, South-Central Minzu University, Wuhan 430074, China; cuiyq@mail.scuec.edu.cn (Y.C.); 202121111111@mail.scuec.edu.cn (R.Y.); baidiwhu@126.com (D.B.); 2 Hubei Key Laboratory of Intelligent Wireless Communications, South-Central Minzu University, Wuhan 430074, China; 3 School of Computer Science, Wuhan University, Wuhan 430074, China; chenxi_wh@sina.com (X.C.); wangdw@whu.edu.cn (D.W.)

**Keywords:** real-scene 3D model, autonomous positioning, image matching, three-dimensional reconstruction

## Abstract

Drones are extensively utilized in both military and social development processes. Eliminating the reliance of drone positioning systems on GNSS and enhancing the accuracy of the positioning systems is of significant research value. This paper presents a novel approach that employs a real-scene 3D model and image point cloud reconstruction technology for the autonomous positioning of drones and attains high positioning accuracy. Firstly, the real-scene 3D model constructed in this paper is segmented in accordance with the predetermined format to obtain the image dataset and the 3D point cloud dataset. Subsequently, real-time image capture is performed using the monocular camera mounted on the drone, followed by a preliminary position estimation conducted through image matching algorithms and subsequent 3D point cloud reconstruction utilizing the acquired images. Next, the corresponding real-scene 3D point cloud data within the point cloud dataset is extracted in accordance with the image-matching results. Finally, the point cloud data obtained through image reconstruction is matched with the 3D point cloud of the real scene, and the positioning coordinates of the drone are acquired by applying the pose estimation algorithm. The experimental results demonstrate that the proposed approach in this paper enables precise autonomous positioning of drones in complex urban environments, achieving a remarkable positioning accuracy of up to 0.4 m.

## 1. Introduction

Drones are extensively employed across diverse domains, including defense, agriculture, disaster management, and military operations, owing to their remarkable efficacy, adaptability, and cost-effectiveness [1]. The utilization of radio technologies such as the Global Navigation Satellite System (GNSS) and cellular network positioning serves as the primary means for drones in urban environments to acquire location information, ensuring both flight safety and operational precision. However, in environments such as urban canyons and building tunnels, signals are prone to obstruction, shielding, or interference, making it difficult to ensure reliable communication and accurate measurements between drones and positioning anchors. The drone’s positioning capability is significantly weakened or even eliminated. Therefore, the development of drones’ autonomous positioning technology in GNSS-denied environments, independent of satellite navigation or wireless communication signals, has emerged as an imperative and prominent research focus to ensure the safe operation of drones [2].

Currently, the majority of research approaches addressing the autonomous positioning of drones employ multi-sensor fusion techniques for positioning. For instance, the integration of GNSS with an Inertial Navigation System (INS) is a common practice. GNSS provides global positioning information [3,4], while the core component of INS, known as the Inertial Measurement Unit (IMU), supplies high-frequency attitude and acceleration data [5]. These data are utilized to compensate for potential drift and interruptions in GNSS signals, thereby enhancing the precision of drone positioning. However, in certain environments, such as densely urban areas and valleys, signals may be obstructed or subject to interference, leading to a decrease in positioning accuracy. Moreover, factors like multipath reflections in urban settings [6], radio interference [7], and adverse weather conditions can also affect the reliability of GNSS. Accurate initial position information is typically required by INS as a starting point; however, inaccuracies in these initial data or accumulated errors during system operation can result in deviations and instability in drone positioning. To address these issues, visual positioning [8] has gradually gained attention.

The technique of visual positioning employs cameras and image processing algorithms to ascertain and monitor the aircraft’s position, orientation, and surrounding environment [9]. Zhang et al. proposed a novel AGV framework called Global Consistent Multi-View Visual–Inertial Fusion (GCMVF-AGV) [10], which utilizes a downward-looking QR visual sensor and a forward-looking inertial sensor for real-time estimation of the AGV’s orientation. Despite the significant advancements achieved in autonomous positioning through the integration of visual images with other sensors and machine learning techniques, visual image positioning technology necessitates substantial computational resources, and it is difficult to meet the positioning requirements in complex environments. Yang et al. proposed a visual–inertial navigation system assisted by polarized light [11]. This integrated system incorporates data from polarized direction sensors, monocular cameras, and micro-inertial measurement units, resulting in a 16.7% reduction in position error and a 23.4% improvement in heading angle accuracy following a series of processing steps. However, the implementation of this system necessitates the utilization of multiple sensors for navigation.

This paper presents a cost-effective and straightforward autonomous drone positioning technology that merges real-scene 3D model data. The implementation of the technology is entirely reliant on the drone itself, eliminating the need for additional nodes or sensors, thereby achieving exceptional positioning accuracy.

A real-scene 3D model is a three-dimensional digital representation based on real-world environments. It is developed through the collection, processing, and reconstruction of data from actual scenes, resulting in virtual 3D models with spatial structures and visual characteristics. These models encompass a variety of environments, such as urban streets, buildings, terrain, and indoor spaces. The real-scene 3D model represents a burgeoning form of infrastructure, with China leading the global forefront in this field. The primary characteristics of real-scene 3D models encompass precise 3D geometric information, abundant terrain elements, accurate spatial relationships, and multi-level detail representation, as emphasized by Academician Deren Li [12]. A stable prior model and precise spatiotemporal reference are provided for wireless measurements in GNSS-denied environments, which can be combined with visual positioning techniques to deliver more accurate and reliable position, orientation, and environmental perception. In this study, a real-scene 3D model is combined with a monocular camera mounted on the drone and image processing algorithms, enabling the drone to achieve real-time capture of its surrounding environment. By leveraging the rich environmental data offered by the 3D model, landmarks, feature points, or textures are identified through image processing algorithms, allowing the drone’s position and orientation to be determined accurately. The proposed approach not only leads to enhanced accuracy and reliability in drone positioning but also facilitates the drone’s perception of its surrounding environment, thereby enabling safer and more efficient autonomous positioning and obstacle avoidance.

The autonomous positioning approach proposed in this paper initially constructs a two-dimensional image dataset and a three-dimensional point cloud dataset by segmenting the real-scene three-dimensional model. Subsequently, real-time images are captured via the onboard monocular camera of the drone, and image matching is performed with the constructed 2D image dataset by employing image-matching algorithms. In accordance with the image matching result, the coarse position information of the drone can be acquired, and the corresponding 3D point cloud data of the real scene within the point cloud dataset can be identified. Furthermore, the captured optical images are subjected to processing, extracting a grayscale image featuring color gradients, which is employed to substitute the depth information in 3D reconstruction and generate real-time 3D point clouds. Meanwhile, voxel downsampling is carried out on the real-scene 3D point cloud data derived from the image-matching results to reduce the complexity of the point cloud data. Principal component analysis (PCA) and the Rodriguez formula are utilized to undertake axis transformation and adaptive size segmentation of the point cloud data. Finally, the reconstructed point cloud data are matched with the processed real-scene 3D point cloud data, and the precise positional information of the unmanned aerial vehicle is obtained through the pose estimation algorithm. The algorithm enables the unmanned aerial vehicle to attain autonomous real-time positioning in circumstances where GNSS signals and other wireless signals are restricted.

## 2. Constructing Datasets

The real-scene 3D model is a comprehensive and spatially structured 3D digital model generated through drone scanning and photogrammetry, enabling the acquisition of precise environmental data. This model exhibits exceptional realism, accuracy, and abundant information content, with data errors meticulously controlled within a margin of 2 cm. Additionally, the model incorporates geographic coordinate information and vivid environmental color details [13,14].

The experimental environment for the proposed autonomous positioning scheme of the drone is situated within an urban building complex. A comprehensive real-scene 3D model of a building is considered excessively voluminous, encompassing data pertaining to the ground, trees, vehicles, and other elements. In accordance with the experimental requirements, image and point cloud datasets are generated from the real-scene 3D model in this study. The point cloud dataset is obtained by segmenting the real-scene 3D model at regular intervals of 1 m, and it is systematically stored using a naming convention that follows the format “location-name-remark-segmented-number.” The image dataset is meticulously aligned with the corresponding point cloud dataset.

The real-scene 3D model is segmented by traversing the point cloud along the *x*, *y*, and *z* axes. Specifically, the *x*-axis is segmented horizontally from left to right, the *y*-axis vertically from front to back, and the *z*-axis perpendicularly from bottom to top. The real-scene 3D model is divided into modules at 1 m intervals to meet the requirements of subsequent point cloud matching algorithms. The overlapping regions between the obtained data ensure continuity and completeness, with the segmentation standard set at 30 m × 30 m × 10 m. To achieve coarse positioning of the drone using image matching algorithms, real-scene 3D image datasets need to be constructed alongside the real-scene 3D point cloud datasets. Specifically, based on the segmented real-scene 3D data, images of the real scene are captured by the drone and saved as corresponding images.

**Figure 1:**
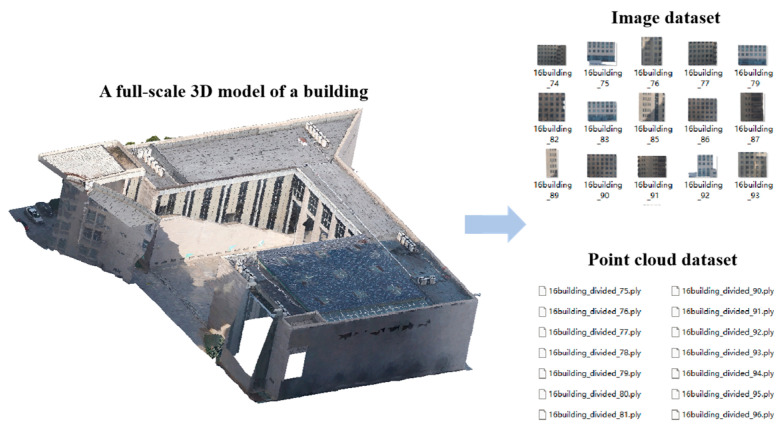
Construction of real-scene 3D point cloud and image datasets.

The establishment of naming conventions ensures the retrieval of corresponding point cloud data through optical image matching, thereby maintaining the association between image and point cloud datasets. For areas with numerical identifiers, such as Building 16, a naming format of “number + building + notes + segmentation number” is employed. For areas lacking numerical identifiers, like playgrounds or art galleries, the format “location name + notes + segmentation number” is applied. The naming convention for images is maintained identically to that of their corresponding point clouds, ensuring a one-to-one correspondence between them. The construction of the point cloud and image datasets through segmentation of the real-scene 3D model is illustrated in Figure 1. As shown in Figure 1, the image dataset displays inconsistencies in resolution, mainly due to some buildings being partially cropped at the edges when using a fixed cutting size. This leads to a limited amount of point cloud data being captured within the cutting range instead of encompassing the complete environmental point cloud data.

## 3. Method

### 3.1. Optical Image Matching

SIFT, Speeded Up Robust Features (SURF) [15], and Oriented FAST and Rotated BRIEF (ORB) [16] are commonly used feature extraction algorithms. Compared to the SURF and ORB algorithms, the SIFT algorithm demonstrates superior feature descriptiveness. Specifically, SIFT outperforms SURF in terms of feature description. Unlike ORB, which is more susceptible to scale and lighting variations and less robust to rotations, SIFT consistently performs well under these conditions. Although the SIFT algorithm has longer processing times, this disadvantage is offset by its superior keypoint matching accuracy, particularly in the context of the extensive image dataset used in our study. Therefore, we chose the SIFT algorithm for our matching tasks.

Given that the size of the image dataset can substantially increase the computational time of the SIFT algorithm, to reduce the matching computation time, we adopted a coarse-to-fine image dataset segmentation strategy. Initially, we matched the drone-captured images with the coarse image dataset, extracted the corresponding fine image dataset based on the matching results, and then matched the images with the fine image dataset. This iterative process effectively minimized the computational time required for image matching.

Table 1 compares the performance of the SIFT algorithm with other algorithms on the same dataset.

From the experimental results above, it can be concluded that the SIFT algorithm outperforms the ORB and SURF algorithms in both runtime and matching accuracy on the dataset constructed for this study.

To perform image matching, it is essential to establish a scale space as the first step. In the SIFT algorithm, a Gaussian convolution kernel is employed for constructing the image’s scale space, facilitating feature detection and description across various scales. By applying Gaussian convolution to the image, a series of Gaussian pyramids with different scales are generated. To improve computational efficiency, the Scale-Invariant Feature Transform algorithm utilizes the Difference of Gaussian (DoG) scale-space [17], representing image feature information by calculating the difference between Gaussian images at adjacent scales.

**Figure 2:**
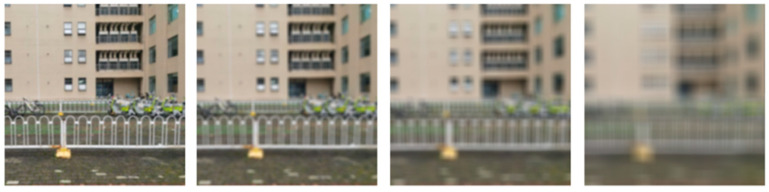
Images under different scale space factors.

The scale space $L\left(x,y,\sigma \right)$ of an image is defined as the convolution of a Gaussian function $G\left(x,y,\sigma \right)$ with a varying scale and the original image, as follows:

(1) $$L\left(x,y,\sigma \right)=G\left(x,y,\sigma \right)*I\left(x,y\right)$$

(2) $$G\left(x,y,\sigma \right)=\frac{1}{2\pi }e^{-\frac{{\left(x-\frac{m}{2}\right)}^{2}+{\left(y-\frac{n}{2}\right)}^{2}}{2\sigma^{2}}}$$

where the symbol ‘∗’ denotes the convolution operation, m,n represent the dimension of the Gaussian template. $\left(x,y\right)$ signifies the position of image pixels and σ is the scale space factor. A smaller value indicates less image smoothing and a corresponding smaller scale. The small scales correspond to the intricate details of the image, while the large scales pertain to its overall features. The effect is illustrated in Figure 2, with the scale increasing from left to right and top to bottom.

**Figure 3:**
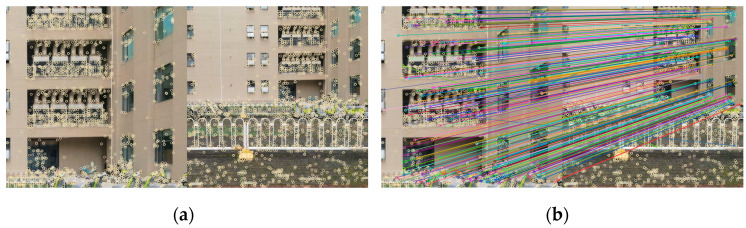
SIFT matching algorithm. (**a**) Effect diagram of feature point extraction; (**b**) Matching effect diagram.

Feature point extraction serves as a prerequisite for image matching. The SIFT algorithm, which is a scale-invariant feature transformation algorithm, enables the extraction of feature points from images across diverse scales and orientations while describing these points. The extracted feature points are illustrated in Figure 3a.

**Figure 4:**
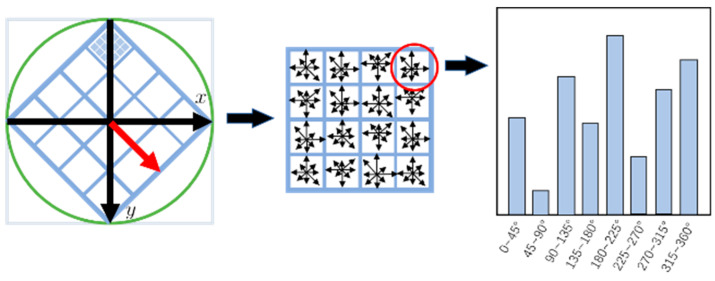
Orientation histogram.

Following feature point extraction, during the feature descriptor generation phase, the SIFT algorithm focuses on each key point and extracts a 16 × 16 pixel region surrounding it. This region is then divided into four 4 × 4 sub-regions, producing a 128-dimensional feature descriptor to represent the image characteristics around the key point. The orientation histogram is displayed in Figure 4.

After generating feature descriptors, separate keypoint descriptor sets are established for the template image (reference image) and the real-time image (observation image). Target recognition is achieved by comparing the keypoint descriptors between the two sets with the similarity of the 128-dimensional keypoint descriptors measured using Euclidean distance.

The three attributes of position, scale, and orientation are retained for each feature point, enabling each keypoint to be described with a vector that remains invariant to changes in illumination, viewpoint, and other external factors.

The descriptor $R_{i}$ in the template image and the key point descriptor $S_{i}$ in the real-time image, the similarity measure $d\left(R_{i}\right.,\left.S_{i}\right)$ between any two descriptors are as follows.

(3) $$R_{i}=\left(r_{i1}\right.,r_{i2},r_{i3},\cdot \cdot \cdot ,\left.r_{i128}\right)$$

(4) $$S_{i}=\left(s_{i1}\right.,s_{i2},s_{i3},\cdot \cdot \cdot ,\left.s_{i128}\right)$$

(5) $$d\left(R_{i}\right.,\left.S_{i}\right)=\sqrt{\sum_{j=1}^{128}\left(r_{ij}-{\left.s_{ij}\right)}^{2}\right.}$$

The matching condition is illustrated by Formula (6), where τ is the set threshold.

(6) $$\frac{d\left(R_{i},\left.S_{j}\right)\right.}{d\left(R_{i},\left.S_{p}\right)\right.}<\tau$$

In Equation (6), the threshold τ is defined as the similarity measure between two feature points. In this study, it is set to 0.7, indicating that two feature points are considered a match if their similarity exceeds 70%. This critical matching threshold was determined through extensive parameter tuning and empirical testing and was ultimately set at 0.7.

The matching effect of the SIFT algorithm is illustrated in Figure 3b.

During the experiment, due to the extensive size of the image dataset, performing matching by traversing the entire dataset, although technically feasible, proved to be time-consuming and failed to meet the desired design requirements. Consequently, an enhanced SIFT algorithm incorporating coarse matching and fine matching is employed, resulting in a significant reduction in matching time and an improvement in operational efficiency.

(7) $$DataSet=\left\{S_{1}\right.,S_{2},\cdot \cdot \cdot ,\left.S_{n}\right\}$$

(8) $$S_{m}=\left\{s_{1}\right.,s_{2},\cdot \cdot \cdot ,s_{x},p_{1},p_{2},\cdot \cdot \cdot ,\left.p_{y}\right\}$$

where DataSet is the master dataset; each element $S_{m}$ in the master dataset contains multiple auxiliary datasets s and multiple images p, until reaching the final layer where no auxiliary datasets are present.

**Figure 5:**
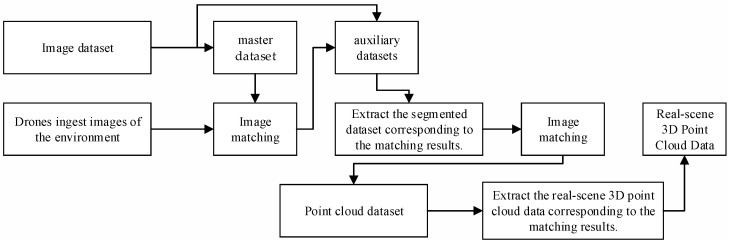
Image Matching Algorithm Processing Framework Diagram.

The algorithm optimization process commences with the establishment of an image dataset comprising a master dataset and multiple auxiliary datasets. For each image in the master dataset, there is a corresponding auxiliary dataset. Each image in the main dataset, which consists of panoramic images from different viewpoints in a specific environment, has a corresponding auxiliary dataset. The auxiliary datasets are generated by segmenting the main images into fixed ranges and processing them individually. The master dataset features images with a wider field of view and larger coverage area, while the auxiliary datasets consist of segmented images from the master dataset, each covering a smaller range. The SIFT algorithm is first applied to match images within the master dataset, with the names of matched images used as the master dataset for the next round of matching. This process repeats until there are no auxiliary datasets remaining in the current folder. The flowchart illustrating the data processing of the optimized image-matching algorithm is presented in Figure 5.

This approach significantly improves code execution efficiency by avoiding the inefficiencies associated with traversing the entire dataset. The comparison of SIFT optimization before and after, using the same dataset, is presented in Table 2. The optimized SIFT algorithm exhibits a 42.4% reduction in running time compared to its unoptimized counterpart.

**Figure 6:**
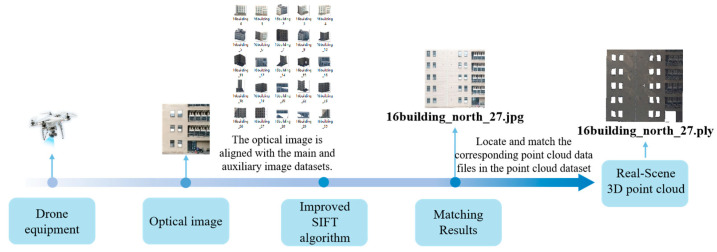
Optical image matching flowchart.

The optimized SIFT algorithm enables rapid and accurate matching of drone images with the constructed image dataset, yielding precise matching results. Leveraging the one-to-one correspondence between the image dataset and the point cloud dataset, these matching results can be utilized to obtain corresponding real-scene 3D point cloud data from the point cloud dataset, reaching coarse positioning. The specific procedure is illustrated in Figure 6.

### 3.2. Image-Based Point Cloud Reconstruction

In this paper, real-time images captured by drones are employed for three-dimensional reconstruction. As the drone’s camera is a monocular camera, direct acquisition of depth information from its raw data is not possible, necessitating the provision of depth information for point cloud reconstruction based on RGB images.

By analyzing the depth images acquired through binocular cameras, it has been found that grayscale images with color gradations can also convey depth information to a certain extent [18]. Therefore, the color value of the image captured by the monocular camera loaded onto the drone is first converted to obtain a grayscale image with color gradations. Subsequently, this grayscale image undergoes inversion and binarization processes. The resulting processed image is then compared with the original image data to identify overlapping regions, which are subsequently cropped. Finally, morphological operations are applied to smoothen the obtained image data before performing three-dimensional reconstruction. The point cloud obtained by this method has roughly identifiable contour information and feature points. During the reconstruction process, the camera’s intrinsic parameter is denoted as K.

(9) $$K=\left[\begin{array}{ccc} f_{x} & 0 & c_{x}\\ 0 & f_{y} & c_{y}\\ 0 & 0 & 1 \end{array}\right]$$

where the horizontal and vertical focal lengths are $f_{x}$ and $f_{y}$, respectively, along with the principal point coordinates are $c_{x}$ and $c_{y}$.

For each pixel $\left(u,\left.v\right)\right.$, the depth value $d\left(u,\left.v\right)\right.$ in the image is converted into corresponding point cloud coordinates $\left(X,Y,\left.Z\right)\right.$.

(10) $$X=\frac{\left(u-\left.c_{x}\right)gd\left(u,\left.v\right)\right.\right.}{f_{x}},Y=\frac{\left(u-\left.c_{y}\right)gd\left(u,\left.v\right)\right.\right.}{f_{y}},Z=\frac{\left(u-\left.c_{z}\right)\right.gd\left(u,\left.v\right)\right.}{f_{z}}$$

The reconstructed point cloud is then segmented and planarized, and the translation transformation matrix Translation and the *Scaling* transformation matrix Scaling are applied to make each point on the same plane to match the segmented real-scene 3D model. Since the reconstructed image is a single monocular image without depth information, the reconstructed image is in the shape of a tetrahedral cone, and each layer of the point cloud is relatively sparse. In order to obtain a point cloud model without thickness and make it denser, the point cloud obtained for the first time must be segmented to obtain a point cloud model with a certain thickness. Then, by translating the points so that each point is on the same plane, a point cloud model that does not contain thickness information while retaining contour information and feature points can be obtained.

(11) $$Translation=\left[\begin{array}{cccc} 1 & 0 & 0 & t_{x}\\ 0 & 1 & 0 & t_{y}\\ 0 & 0 & 1 & t_{z}\\ 0 & 0 & 0 & 1 \end{array}\right],Scaling=\left[\begin{array}{cccc} S_{x} & 0 & 0 & 0\\ 0 & S_{y} & 0 & 0\\ 0 & 0 & S_{z} & 0\\ 0 & 0 & 0 & 1 \end{array}\right]$$

where $t_{x}$, $t_{y}$, $t_{z}$ are the translation amounts and $S_{x}$, $S_{y}$, $S_{z}$ are the scaling factors.

There is a certain coordinate axis deviation between the generated point cloud and the segmented model. To facilitate subsequent point cloud matching, the reconstructed point cloud model and the segmented real-scene 3D model are aligned on a common coordinate axis using rotation matrices R and T.

(12) $$R=\left[\begin{array}{ccc} \cos\left(\theta \right) & 0 & \sin\left(\theta \right)\\ 0 & 1 & 0\\ -\sin\left(\theta \right) & 0 & \cos\left(\theta \right) \end{array}\right],T=\left[\begin{array}{ccc} -1 & 0 & 0\\ 0 & -1 & 0\\ 0 & 0 & 1 \end{array}\right]$$

**Figure 7:**
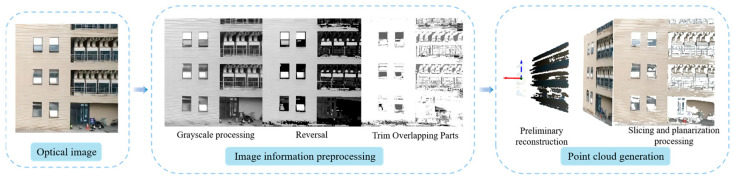
Three-dimensional reconstruction flowchart.

The flowchart illustrating the real-time 3D point cloud reconstruction is depicted in Figure 7.

### 3.3. Three-Dimensional Point Cloud Matching

The point cloud data derived from the segmentation of the real-scene three-dimensional model possess a relatively high density. To further improve the positioning accuracy and ascertain the positional information of the drone, the real-scene 3D point cloud data extracted based on the image matching outcomes are subjected to point cloud matching with the real-time 3D point cloud data generated by the reconstruction from the images captured by the UAV, thereby obtaining precise matching results.

The centroid of the point cloud data of the real-scene three-dimensional model can be obtained by calculation based on Formula (13).

(13) $$centroid=\frac{1}{n}\sum_{i=1}^{n}po\mathrm{int}s_{i}$$

where centroid is the centroid of the point cloud, points is a set of n points, and $po\mathrm{int}s_{i}$ is the i-th point in the set.

By subtracting the centroid of the acquired point cloud from the real-scene 3D point cloud data, the point cloud can be translated to align with the coordinate origin while simultaneously recording the translation magnitude.

The accuracy of the real-scene 3D model is within a range of 2 cm. The model encompasses a substantial amount of point cloud data. To optimize algorithmic efficiency, it is necessary to appropriately reduce the density of the point cloud while preserving the utmost building outline information.

The algorithm in this paper employs voxel downsampling [19]. First, the point cloud space is partitioned into a cubic grid with a given voxel size as the side length. Subsequently, a representative point located at the center of each voxel is selected, and a nearest neighbor search is conducted using a KD tree to extract color information from nearby points within the voxel size range. This extracted information is then utilized to compute the average color value, which subsequently becomes assigned as the color attribute for the central point of that particular voxel. Finally, all the retained voxel center points are combined into a new, lower-density point cloud.

**Figure 8:**
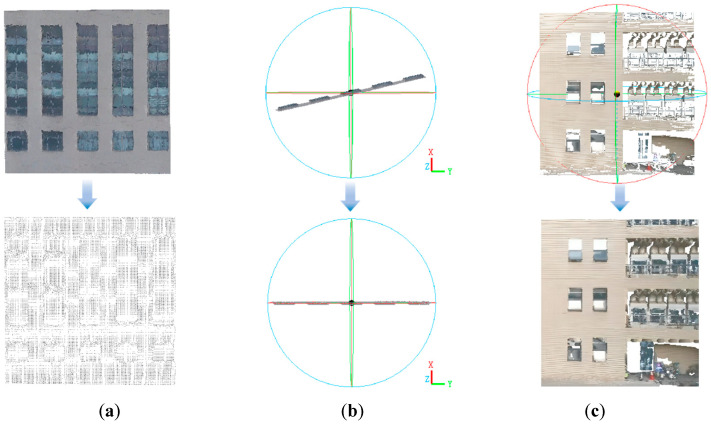
Schematic diagram of some steps of 3D point cloud matching. (**a**) illustrates the schematic of voxel-based downsampling. (**b**) presents a schematic illustration of rotating the point cloud to the YOZ plane. (**c**) displays the resultant image obtained from transforming the point cloud.

The voxel downsampling technique effectively preserves the overall structure and features of the point cloud. Therefore, compared to alternative methods like random sampling, it maintains the shape of the original data as much as possible. Experimental verification demonstrates that when the voxel size is 0.1 m, the algorithm rate can be increased as much as possible while ensuring matching accuracy, as illustrated in Figure 8a.

The experimental environment for drone positioning in this study is situated within an urban building complex. The majority of buildings consist of walls, and the real-scene 3D model of the wall is a planar point cloud. To facilitate subsequent adaptive cutting algorithms, it is necessary to rotate the point cloud onto a plane aligned with the coordinate axis. The algorithm in this paper selects the YOZ plane.

The algorithm presented in this paper utilizes the principal component analysis (PCA) method [20] to compute the normal vector of the planar point cloud. The data are first standardized by calculating the mean $\mu_{j}$ and standard deviation $\sigma_{j}$ for each feature j. Then, the eigenvalue $x_{ij}$ in each sample i is normalized using $z_{ij}$. Finally, the covariance matrix is computed. Given n samples and m features, the data matrix is X, and the elements of the covariance matrix C are $C_{jk}$.

(14) $$Z_{ij}=\frac{x_{ij}-\mu_{j}}{\sigma_{j}},C_{jk}=\frac{1}{n-1}\sum_{i=1}^{n}\left(z_{ij}-{\bar{z}}_{j}\right)\left(z_{ik}-{\bar{z}}_{k}\right)$$

where ${\bar{z}}_{j}$ and ${\bar{z}}_{k}$ are the means of feature j and feature k, respectively.

Finally, the covariance matrix C is subjected to eigenvalue decomposition Cv=λv in order to obtain the eigenvalue $\lambda_{1},\lambda_{2},\cdot \cdot \cdot ,\lambda_{k}$ and its corresponding eigenvector $v_{1},v_{2},\cdot \cdot \cdot ,v_{m}$.

The normal vector is commonly defined as the principal component corresponding to the direction of minimum variance in the distribution of data. In a point cloud, the principal directions of the data usually correspond to the normal vectors of the surface. We only need to calculate the covariance matrix of the data and extract the last principal component (that is, the eigenvector corresponding to the minimum eigenvalue) as the normal vector $n_{1}$ of the point cloud.

Define the normal vector of the YOZ plane as $n_{2}=\left[1,0,0\right]$, and employ the obtained normal vector of the target point cloud to calculate the rotation matrix using the Rodriguez formula [21]. The rotation $axis=n_{1}\times n_{2}$ is first determined by calculating it through two vectors, followed by the calculation of the rotation angle θ.

(15) $$\theta =\arccos\left(\frac{n_{1}\cdot n_{2}}{\left\|n_{1}\right\|\cdot \left\|n_{2}\right\|}\right)$$

Finally, the calculation of the rotation matrix R is performed based on the Rodriguez Formula (16).

(16) $$R=I+\sin\left(\theta \right)\cdot K+\left(1-\cos\left(\theta \right)\right)\cdot K^{2}$$

where *I* is the identity matrix, and the matrix *K* is defined as Formula (17).

(17) $$K=\left[\begin{array}{ccc} 0 & -axis_{z} & axis_{y}\\ axis_{z} & 0 & -axis_{x}\\ -axis_{y} & axis_{x} & 0 \end{array}\right]$$

where $axis_{x}$, $axis_{y}$, $axis_{z}$ are the components of the rotation axis.

The target point cloud can be rotated to the YOZ plane by calculating the rotation matrix. The data presented in Figure 8b demonstrate this process.

In order to achieve precise positioning, it is necessary to adaptively segment the environmental point cloud data corresponding to the optical image matching results after undergoing translation and rotation preprocessing.

When the drone captures images, the shooting instrument will display the distance between the drone and the building. The distance can be used to calculate the ratio between the three-dimensional reconstruction point cloud and the actual dimensions of the photographed building. Experimental analysis demonstrates a nearly linear relationship between distance D and ratio P, as depicted in Formula (18).

(18) P=k∗D+b

The values of k and b are obtained through specific experimental data analysis. When the algorithm is actually applied, after the drone captures an image, the image and the distance information D to the photographed building are transmitted to the self-positioning system. The image is first reconstructed in three dimensions, and the length and width of the reconstructed planar point cloud are obtained. The ratio *P* is then calculated based on the distance D. Finally, the actual size of the photographed building can be obtained by multiplying the two data points, which are the length and width of the adaptive segmentation point cloud.

Considering the algorithm’s efficiency and accuracy, the segmentation interval of this algorithm is set to 0.2 m. The segmentation algorithm is similar to the algorithm for segmenting the real-scene 3D model to construct a point cloud dataset, with the exception that the resulting segmented point cloud data are stored in a list instead of being saved as a separate point cloud file.

All the point cloud data for segmenting in the obtained list is converted into image format to form a dataset of cutting point cloud images, and the three-dimensional reconstruction point cloud is also transformed into an image.

First, select the coordinates of the point cloud in the *y* and *z* directions to draw a scatter plot; then, set the coordinate axis range by finding the maximum and minimum values in the *y* and *z* directions. Finally, adjust the image edge so that the image edge is closely around the drawn figure, save the drawn image in JPG format, and unify the naming format. An example of this conversion is illustrated in Figure 8c.

By employing the aforementioned algorithm, we obtain a three-dimensional reconstruction point cloud image and a set of segmented point cloud images. Subsequently, we employ the SIFT feature matching algorithm to match the three-dimensional reconstruction point cloud image and the segmented point cloud image set for precise positioning. Finally, we return the best matching image file name.

### 3.4. Pose Positioning

The best matching image file name returned by the 3D point cloud matching algorithm can be used to match the corresponding point cloud data according to the image file name. The rotation matrix derived from the aforementioned algorithm is employed for computing the inverse matrix of said rotation matrix. The calculation formula is presented as Formula (19).

(19) $$R^{-1}=\frac{1}{\det\left(R\right)}\cdot adj\left(R\right)$$

where *R* is the rotation matrix obtained from point cloud matching, *adj*(*R*) represents the adjoint matrix of matrix *R*, and *det*(*R*) represents the determinant of matrix *R*.

The point cloud data before rotation can be restored by operating the point cloud data through the inverse matrix; adding the translation amount subtracted by the point cloud matching algorithm, the final matched set of point cloud data can be restored in the real-scene 3D model, which is also the real coordinates of the location of the image in the real-scene 3D coordinate system. By calculating the centroid of this restored point cloud data, a set of coordinates can be derived.

Similarly, the drone should capture an image of the building on the opposite side from the same location and apply the aforementioned algorithm for the same processing to obtain another set of coordinates. Extract the *x*-axis value from the first set of coordinates and then acquire the *y*-axis value from the other set of coordinates. These newly obtained coordinates represent accurate positional information of the drone within the point cloud coordinate system of a real-scene 3D model.

**Figure 9:**
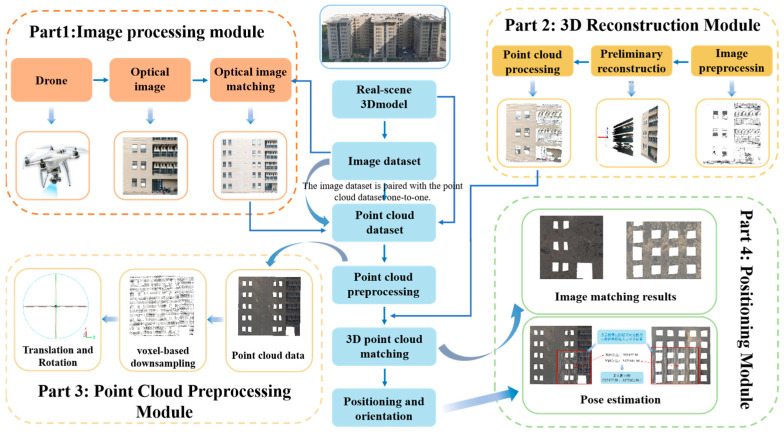
Overall framework of the self-positioning scheme.

The comprehensive block diagram illustrating the proposed self-positioning method is presented in Figure 9, which comprises four main components: First, the image processing module, which employs drones to capture environmental images and matches them with image datasets to generate matching results, thereby facilitating subsequent experiments. Second, the 3D reconstruction module uses the captured images to perform 3D reconstruction. Third, the point cloud processing module extracts corresponding environmental point cloud data from a point cloud dataset based on the image-matching results and processes it accordingly. Finally, the localization module aligns the 3D reconstructed point cloud with the environmental point cloud and determines the precise location of the drone through pose estimation.

## 4. Discussion

### 4.1. Experimental Scene and Equipment

The self-positioning method proposed in this paper was experimentally verified by conducting experiments involving drones and real-scene 3D model datasets, thereby obtaining specific positioning results.

**Figure 10:**
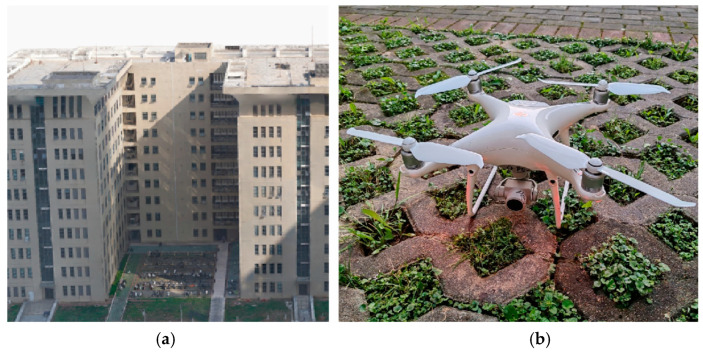
Experimental scene and equipment. (**a**) Experimental scenario. (**b**) A diagram of a drone.

The experimental site is situated in the open-air parking lot behind the university’s teaching building, as depicted in Figure 10a. The utilized drone equipment corresponds to the DJI Phantom 4 Pro V2.0 model, as illustrated in Figure 10b. The drone’s fuselage dimensions (excluding propellers) measure approximately 220 × 220 × 242 mm, with a diagonal wheelbase of 350 mm and a total weight (including battery and camera) of around 1388 g. It exhibits a maximum flight speed of 72 km/h under windless conditions and demonstrates stable flight capabilities even in the absence of GNSS signals.

### 4.2. Experimental Process

**Figure 11:**
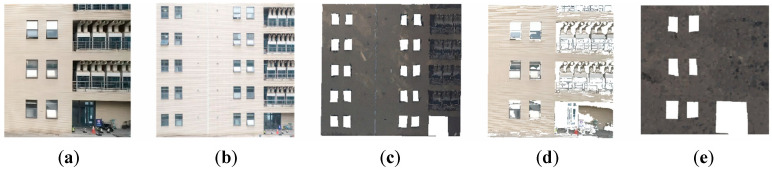
Front image processing flow. (**a**) the frontal photos taken by the drone. (**b**) the initial positioning result image. (**c**) the point cloud file corresponding to the image matching result. (**d**) the 3D reconstruction point cloud. (**e**) the point cloud matching result.

In the verification experiment, a single-eye camera mounted on a drone was employed to capture frontal and lateral images of the experimental scene. These two images were subsequently inputted into the self-positioning system for a series of processing steps, achieving autonomous positioning of the drone. Following the processing of frontal and lateral images captured by the drone, matching results aiding in position information estimation were obtained. Schematic diagrams illustrating each step during image processing are presented in Figure 10 and Figure 11.

Figure 11a presents the frontal image captured by the drone according to the experimental scene. Figure 11b illustrates the matching result between the drone-captured image and the image datasets. The specific processing workflow involves initially matching the image with the primary dataset, followed by a secondary match with the corresponding auxiliary dataset based on the initial results. Figure 11c displays the three-dimensional point cloud derived from the image-matching process. Figure 11d shows the point cloud generated from the three-dimensional reconstruction of the drone-captured image, following the same procedure as described in Section 3 for image-based point cloud reconstruction. Figure 11e presents the matching result between the environmental point cloud and the reconstructed point cloud.

Based on the analysis of the matching results presented in Figure 11b, the extracted environmental 3D point cloud encompasses a broader area compared to the reconstructed point cloud, which only occupies a portion of the environmental point cloud. To address this discrepancy, we segmented the environmental point cloud according to the dimensions of the reconstructed point cloud and employed a cutting interval of 0.5 m. Using this approach, we successfully achieved the matching results displayed in part (e) of Figure 11 after aligning the reconstructed image with the segmented images.

**Figure 12:**
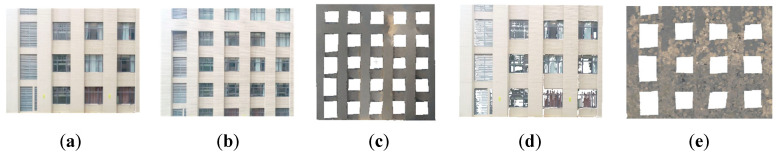
Surface image processing flow. (**a**) the lateral photos taken by the drone. (**b**) the initial positioning result image. (**c**) the point cloud file corresponding to the image matching result. (**d**) the 3D reconstruction point cloud. (**e**) the point cloud matching result.

Figure 12a presents the lateral image captured by the drone. Figure 12b represents the matching outcome obtained by matching the collected image with the image dataset. Figure 12c depicts the corresponding real-scene 3D point cloud data file extracted based on the image-matching results from the real-scene 3D point cloud dataset. Figure 12d displays the result of the 3D reconstruction of the lateral image captured by the drone. Figure 12e shows the matching results of the point cloud matching between the environmental point cloud data extracted corresponding to the image matching results and the 3D point cloud acquired through 3D reconstruction of the image.

Furthermore, for the environmental 3D point cloud extracted from the matching results presented in Figure 12b, we applied the same processing workflow as described in Figure 11, resulting in the matching outcomes shown in Figure 12e.

**Figure 13:**
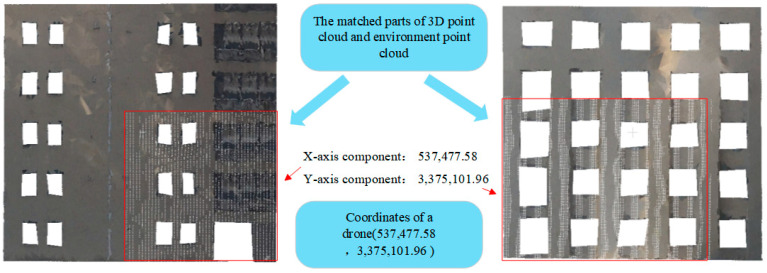
Pose estimation to determine the coordinates of the drone.

By employing the pose estimation algorithm, a set of coordinates (537,477.58, 3,375,078.06, 3.72) can be computed based on the point cloud data corresponding to the 3D point cloud matching outcomes from the frontal image. And the x-axis component of the coordinates is taken as the x-axis component of the coordinates of the drone’s location. At the same time, another set of coordinates (537,458.74, 3,375,101.96, 3.72) can be calculated based on a set of point cloud data corresponding to the 3D point cloud matching results of the side image, and the y-axis component of the coordinates is taken as the y-axis component of the coordinates of the drone’s location, as shown in Figure 13.

From the above experiment, the drone’s self-positioning system accurately determined the position of the drone in the real-scene 3D coordinate system as (537,477.58, 3,375,101.96, 3.72) based on the front and side images captured by the drone. And the positioning time is 346.57 ms.

Throughout the experiment, the reference coordinates of the real-scene 3D coordinates are very large, which brings challenges to data processing. To address this issue, the size of the real-scene 3D point cloud data is adjusted by subtracting the reference coordinate values of the real-scene 3D model from it. In this way, the final positioning result is added to the reference coordinates, which facilitates the recovery of the actual geographic coordinates.

**Figure 14:**
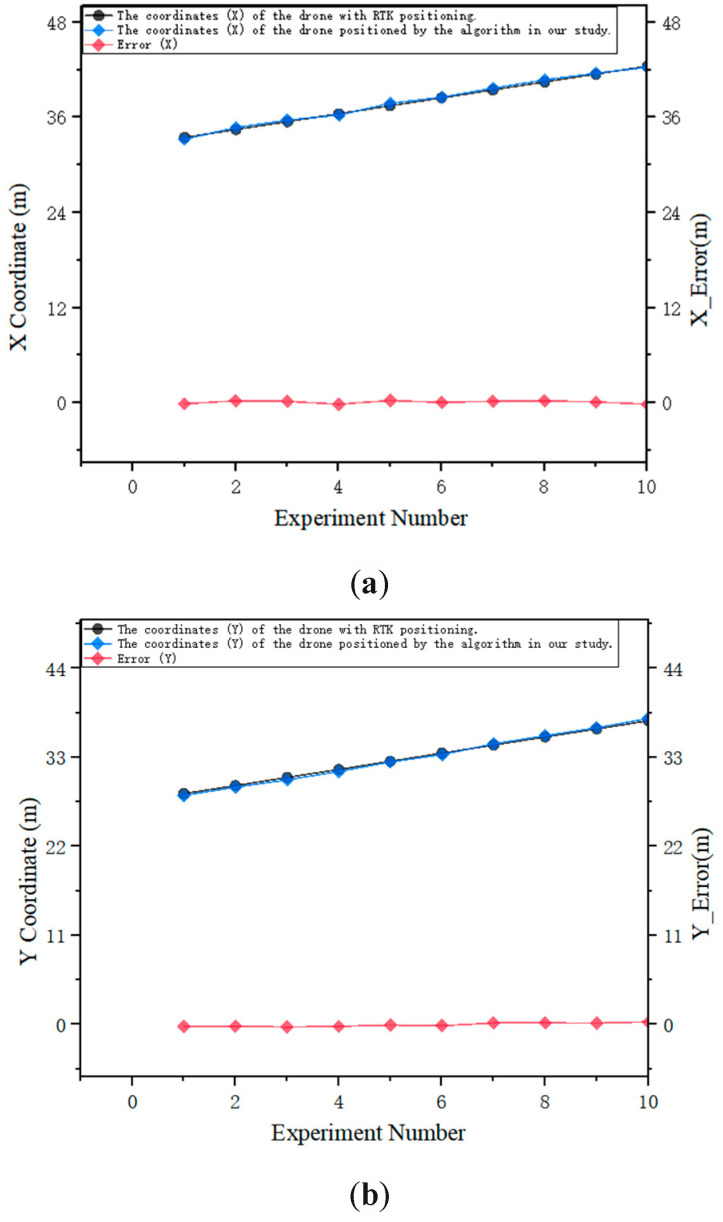
Drone coordinates and errors of RTK and algorithm positioning in *X* and *Y* dimensions. (**a**) the comparison between the X coordinate values calculated by the drone using the algorithm presented in this paper and those obtained through RTK positioning, along with the relative error between the two. (**b**) the comparison between the Y coordinate values calculated by the drone using the algorithm presented in this paper and those obtained through RTK positioning, along with the relative error between the two.

To verify the accuracy of the algorithm in this paper, a group of photos is captured every time the drone moves one meter left and right, as well as one meter forward and backward. The coordinates of the drone are calculated in real-time using the self-positioning algorithm in this paper. While the drone is capturing images, the laser rangefinder is utilized to record the precise distance between the drone and the wall. Subsequently, high-precision positioning of the drone’s image location is achieved using real-time kinematic (RTK) technology based on the real-time dynamic carrier phase difference. This experimental approach enables the attainment of centimeter-level positioning accuracy as actual coordinates for drone positioning. The positioning coordinates of the two are ultimately compared within the same coordinate system. The positioning coordinates and errors of the RTK and proposed algorithm in the *X* and *Y* dimensions at ten different positions of the drone are depicted in Figure 14.

Simultaneously, to validate the real-time performance of the algorithm proposed in this study, the positioning time (Pos_time) is automatically recorded when employing the algorithm for drone positioning. The corresponding results are presented in Table 3.

The Formula (20) presents the calculation of the drone positioning error, Error. Table 3 displays the drone positioning error data for the aforementioned ten datasets.

(20) $$Error=\sqrt{{\left(X\_Error\right)}^{2}+{\left(Y\_Error\right)}^{2}}$$

The results presented in Table 3 demonstrate that the proposed algorithm achieves a positioning accuracy of 0.4 m, with an average positioning time of 345.49 ms.

### 4.3. Results Analysis

The accuracy of the experimental positioning results is evaluated using the depth root mean square error (DRMS) and mean radial spherical error (MRSE) [22], while the real-time performance of the positioning results is assessed based on the average localization time $\bar{T_{loc}}$. The calculation formula is provided as follows:

(21) $$DRMS=\sqrt{\frac{1}{N}\sum_{i=1}^{n}d_{i}^{2}},MRSE=\sqrt{\frac{1}{N}\sum_{i=1}^{n}\left(x_{i}^{2}+y_{i}^{2}+z_{i}^{2}\right)}$$

where N is the number of observations and $d_{i}$ is the error in the x dimension of the i-th observation. The errors in three directions of the i-th observation are denoted as $x_{i},y_{i},z_{i}$, while $T_{loc}^{\left(i\right)}$ represents the duration of positioning in the i-th experiment.

**Figure 15:**
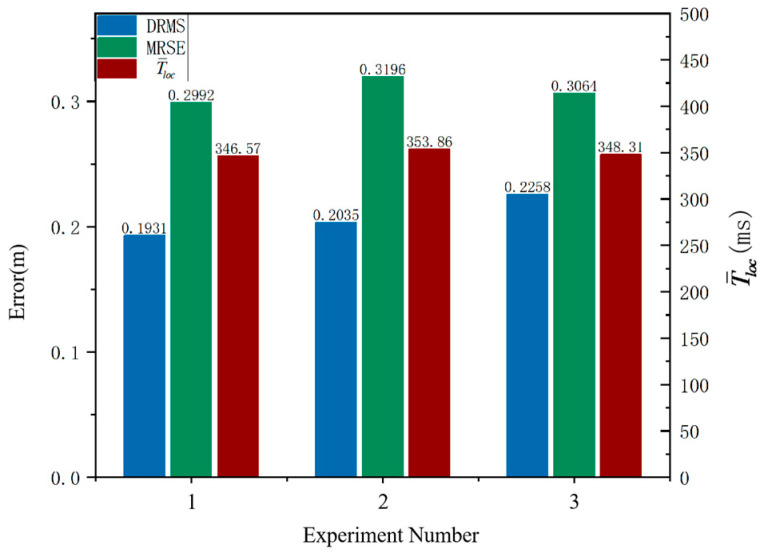
Positioning error and positioning time results of multiple groups of experiments.

Three sets of experiments are conducted in this experimental scenario, with ten data groups obtained for each set. The DRMS and MRSE of the positioning results, as well as ${\bar{T}}_{loc}$ values, are calculated based on each data group in Figure 15.

As shown in Figure 14, in this experimental scenario, the average DRMS is 0.2075 m, the average MRSE is 0.3084 m, and the positioning accuracy is high; the average ${\bar{T}}_{loc}$ is 349.58 ms, and the real-time positioning is commendable.

The autonomous positioning approach presented in this paper is capable of offering precise positioning information for the drone. Furthermore, the solution proposed in this paper offers clear advantages over most current self-positioning methods regarding sensor count. Additionally, the dataset constructed from the real-scene model does not require pre-training within the algorithm and can be directly utilized for algorithmic processing. Table 4 presents a comparison of the relevant parameters of the autonomous positioning scheme proposed in this paper with those of other existing schemes. Reference [23] utilized a rangefinder radar and an infrared camera to achieve low-altitude positioning of drones. Initially, the infrared camera captured video footage of the target environment, which was subsequently segmented into individual frames for analysis. The YOLOv7 model was then employed to detect the presence of drones in these frames. Finally, the precise location of the drone was estimated using data from the rangefinder radar. Additionally, Reference [24] investigated a GNSS/INS hybrid navigation system augmented by LiDAR. When GNSS signals were unavailable, the system switched to INS/LiDAR mode for accurate target positioning. Experiments were conducted in an experimental environment identical to that described in this paper to validate the effectiveness of both methods.

## 5. Conclusions

To address the challenge of autonomous positioning for drones in complex urban environments where GNSS and other radio signals used for positioning are unavailable, this paper proposes a real-scene 3D model-driven autonomous positioning method. This approach enables high-precision, fully autonomous positioning of unmanned systems without relying on GNSS. The autonomous positioning approach proposed in this paper initially constructs a two-dimensional image dataset and a three-dimensional point cloud dataset by segmenting the real-scene three-dimensional model. Subsequently, real-time images are captured via the onboard monocular camera of the drone, and image matching is performed with the constructed 2D image dataset by employing image-matching algorithms. In accordance with the image matching result, the coarse position information of the drone can be acquired, and the corresponding 3D point cloud data of the real scene within the point cloud dataset can be identified. Furthermore, the captured optical images are subjected to processing, extracting a grayscale image featuring color gradients, which is employed to substitute the depth information in 3D reconstruction and generate real-time 3D point clouds. Meanwhile, voxel downsampling is carried out on the real-scene 3D point cloud data derived from the image-matching results to reduce the complexity of the point cloud data. Principal component analysis (PCA) and the Rodriguez formula are utilized to undertake axis transformation and adaptive size segmentation of the point cloud data. Finally, the reconstructed point cloud data are matched with the processed real-scene 3D point cloud data, and the precise positional information of the unmanned aerial vehicle is obtained through the pose estimation algorithm. Compared with other positioning methods, the proposed autonomous positioning method achieves a positioning accuracy of 0.4 m in complex environments, eliminating the need for positioning anchors or other nodes and reducing dependence on sensors.

## Figures and Tables

**Table 1:** Comparison of SIFT with other matching algorithms.

Algorithm Type	Run Time	Matching Accuracy
SIFT Algorithm	124 ms	99%
ORB Algorithm	132 ms	99%
SUFT Algorithm	157 ms	80%

**Table 2:** Comparison of SIFT algorithm before and after optimization.

	Run Time	Matching Accuracy
Before optimization	224 ms	precise
After optimization	124 ms	precise

**Table 3:** Drone positioning error and positioning timetable.

	1	2	3	4	5	6	7	8	9	10
**Error (m)**	0.264	0.347	0.389	0.311	0.322	0.193	0.266	0.328	0.178	0.326
**Pos_time (ms)**	346.57	351.42	344.38	333.71	349.25	352.64	337.73	345.47	344.58	349.19

**Table 4:** Comparison with other solutions.

	The Proposed Algorithm	Reference [23]	Reference [24]
Data Preprocessing	unnecessary	necessary	necessary
Number of Sensors	1	2	4
Positioning Accuracy	0.4 m	0.5 m	1.66 m

## Data Availability

The datasets involved in this study is a private one and is not publicly archived at present. This decision is based on considerations of privacy.
